# Comparative Analysis of the Gut Microbiota of Mongolian Gazelle (*Procapra gutturosa*) Under Fragmented Habitats

**DOI:** 10.3389/fmicb.2022.830321

**Published:** 2022-03-09

**Authors:** Lupeng Shi, Xiufeng Yang, Huashan Dou, Tianshu Lyu, Lidong Wang, Shengyang Zhou, Yongquan Shang, Yuehuan Dong, Honghai Zhang

**Affiliations:** ^1^College of Life Sciences, Qufu Normal University, Qufu, China; ^2^Hulunbuir Academy of Inland Lakes in Northern Cold & Arid Areas, Hulunbuir, China

**Keywords:** *Procapra gutturosa*, gut microbiota, metagenomic, fragmented habitats, naphthalene degradation

## Abstract

The existence of man-made facilities such as pasture fences makes the grassland ecosystem fragmented and endangers the survival of local wild animals. The Mongolian gazelle is highly sensitive to hunting and habitat destruction, and is one of the most threatened artiodactyls in Eurasia. It provides a critical model to studying gut microbiota under fragmented habitats. Therefore, we applied metagenomics sequencing to analyze the gut microbiota communities and functions of Mongolian gazelle under fragmented habitats. The results demonstrated that there were no significant differences in gut microbial communities between the different groups at both the phylum and genus level. The functional analyses showed that the Mongolian gazelle in fragmented habitat had a stronger ability to degrade naphthalene, but their ability to absorb carbohydrates was weaker. This study provided fundamental information about the gut microbiota of Mongolian gazelle, and we recommend reducing habitat fragmentation to better protect the Mongolian gazelle.

## Introduction

The gut microbiota of mammals is frequently affected by many factors, including dietary choices, phylogeny, and environmental changes (Turnbaugh et al., 2009; Yatsunenko et al., 2012; Schnorr et al., 2014; Clemente et al., 2015). The diverse and extremely complex gut microbiota benefits host in many ways, such as synthesizing vitamins, stimulating immune responses, and performing metabolic functions that the host cannot perform (Backhed et al., 2005; Cerf-Bensussan and Gaboriau-Routhiau, 2010; Leblanc et al., 2013; Crespo-Piazuelo et al., 2018; Nagarajan et al., 2018). Ruminants rely on the gut microbiota to harness energy *via* the fermentation of dietary material (Thoetkiattikul et al., 2013). Thus, the gut microbiota plays an important role in the nutritional ecology of ruminants (Hu X. et al., 2018). Recent studies indicate that anthropogenic disturbance can cause major changes in the composition of the gut microbiota (Barelli et al., 2020). For instance, populations of both the black howler monkey and Udzungwa red colobus have been shown to have lower gut microbiota diversity in fragmented habitats compared with intact habitats (Amato et al., 2013; Barelli et al., 2015). Given that the gut microbiota plays a crucial role in host health (Fung et al., 2017), understanding the effects of anthropogenic disturbance on host’s gut microbiota is critical for developing effective conservation strategies for endangered species (Stumpf et al., 2016).

The Mongolian gazelle (*Procapra gutturosa*) is a unique herbivorous animal on the steppe habitats in Eurasia and was listed as a Category I species in the China’s Red List of Biodiversity: Vertebrates (Zhang et al., 2014; Jiang et al., 2021). The species was once widespread in Mongolia, northern China, and southeastern Siberia (Wang et al., 1997). A previous study of Mongolian gazelle has focused on behavioral characteristics, feeding habits, and migration (Li et al., 1999; Leimgruber et al., 2001; Odonkhuu et al., 2009). Mongolian gazelle population in Hulun Lake National Nature Reserve is affected by anthropogenic disturbance due to the existence of human facilities such as grassland fence, and the habitat is fragmented. Affected by the fragmented environment, the Mongolian gazelle formed a locally isolated population in this area, which greatly increased the risk of its local extinction. In contrast, the Mongolian gazelle population in the China–Mongolia border area has been virtually unaffected by anthropogenic disturbance, and the habitat is complete. Therefore, we speculate that the composition and function of gut microbiota would be altered in populations restricted to fragmented habitats.

The present study aimed to explore gut microbial diversity and function of Mongolian gazelle populations under fragmented habitats by using the metagenomic sequencing. This research will improve the understanding of the differences in gut microbiota of the Mongolian gazelle in different conditions and provide a scientific reference for the protection and management of this species.

## Materials and Methods

### Sample Collection

Samples were collected from China–Mongolia border area and Hulun Lake National Nature Reserve. Detailed information for all samples is shown in Supplementary Table 1. Eight microsatellite loci (OArFCB304, SPS115, TGLA68, IOBT395, PZE114, MNS72, BM1341, and MB066) were used to identify individuals (Buchanan and Crawford, 1993; Reed and Beattie, 2001; Chu et al., 2002; Motavalian et al., 2002; Lv et al., 2008; Fend et al., 2010; Nijman et al., 2010). This identification allows all alleles identical or only one mismatch (Ning et al., 2018). It was identified that the fecal samples of H group (*n* = 4) and B group (*n* = 5) were all from different individuals. Detailed information for microsatellite loci of Mongolian gazelle is shown in Table 1. During the sampling period, the ambient temperature was approximately −30°C to ensure the quality of DNA from gut microbiota. Fecal samples were placed in sterile containers and stored at −80°C until DNA extraction.

**TABLE 1:** Detailed information for microsatellite loci of Mongolian gazelle.

Sample	OArFCB304		SPS115		TGLA68		IOBT395		PZE114		MNS72		BM1341		MB066	
H1	141	145	254	256	81	81	90	98	93	93	166	166	118	120	100	100
H2	141	145	252	254	81	111	90	94	93	111	164	166	120	120	100	100
H3	131	141	254	258	81	81	82	94	91	91	164	166	120	120	100	100
H4	141	145	252	252	81	81	88	94	93	93	166	166	118	120	100	100
B1	133	141	250	252	81	81	82	88	91	91	164	166	116	126	100	100
B2	131	145	252	252	81	81	90	94	93	93	164	166	118	122	96	100
B3	137	151	254	254	81	81	100	106	93	93	164	166	110	116	98	98
B4	145	147	254	256	81	81	94	94	93	93	164	166	116	132	98	100
B5	131	135	252	256	81	81	92	92	93	111	166	166	118	118	98	124

### DNA Extraction, Library Construction, and Metagenomics Sequencing

According to the manufacturer’s recommendations, metagenomic DNA was extracted from the internal part of feces using QIAamp Fast DNA Stool Mini Kit (Qiagen, Germany).

Extracted DNA was monitored using 1% agarose gels to determine degradation degree and potential contamination. We measured the concentration of DNA with Qubit dsDNA Assay Kit in Qubit 2.0 Flurometer (Life Technologies, CA, United States). Only samples that meet the following criteria were used to construct the library: (1) OD value is between 1.8 and 2.0; (2) DNA contents above 1 μg.

The qualified DNA was fragmented to a size of 350 bp using sonication. DNA fragments were end-polished, A-tailed, and ligated with the full-length adaptor for sequencing and further PCR amplification. The PCR products were purified with AMPure XP system. Next, we analyzed the size distribution of libraries by Agilent 2100 Bioanalyzer and quantified libraries by real-time PCR. According to the manufacturer’s instructions, we performed the clustering of the index-coded samples on a cBot Cluster Generation System. After cluster generation, Illumina HiSeq platform was used to sequence the library preparations and paired-end reads were generated.

### Sequencing Results Pretreatment and Metagenome Assembly

Readfq was used to delete the reads in raw data with quality threshold value ≤38, N base length ≥10 bp, and overlap length ≥15 bp. The reads from host origin were filtered by Bowtie2.2.4 software with the following parameters: –end-to-end, –sensitive, -I 200, -X 400 (Karlsson et al., 2012, 2013). Clean data were acquired for subsequent analysis.

Metagenome assembly was performed using SOAPdenovo software (Luo et al., 2012). The parameters were as follows: -d 1, -M 3, -R, -u, -F, -K 55 (Scher et al., 2013; Qin et al., 2014; Brum et al., 2015; Feng et al., 2015). Scaffolds were interrupted from N connection to obtain Scaftigs without N (Mende et al., 2012; Nielsen et al., 2014; Qin et al., 2014). To acquire unused PE reads, we mapped all samples’ clean data to each scaffold respectively with Bowtie2.2.4 software. The parameters were as follows: –end-to-end, –sensitive, -I 200, -X 400 (Qin et al., 2014).

### Gene Prediction, Taxonomy, and Functional Annotations

Open reading frame (ORF) predictions for Scaftigs (≥500 bp) were produced using MetaGeneMark software (Zhu et al., 2010; Karlsson et al., 2012, 2013; Mende et al., 2012; Nielsen et al., 2014; Oh et al., 2014), and the length information (<100 nt) from the predicted result was filtered (Qin et al., 2010, 2014; Li et al., 2014; Nielsen et al., 2014; Zeller et al., 2014). CD-HIT software was adopted to remove redundancy and obtain initial gene catalog with the following parameters: -c 0.95, -G 0, -aS 0.9, -g 1, -d 0 (Li and Godzik, 2006; Fu et al., 2012; Zeller et al., 2014; Sunagawa et al., 2015). We mapped the clean data of each sample to initial gene catalog with Bowtie2.2.4 and calculated the number of reads mapped in each sample. The parameters were as follows: –end-to-end, –sensitive, -I 200, -X 400 (Li et al., 2014; Qin et al., 2014). To acquire unigenes for subsequent analysis, the genes with the number of reads ≤2 were filtered (Qin et al., 2012; Li et al., 2014). According to the length of genes and mapped reads, we calculated the relative abundance of the unigenes.

We blasted the unigenes to the sequences of bacteria, fungi, archaea, and viruses from the NR database of NCBI using DIAMOND software (Buchfink et al., 2015). According to the result with *e* value ≤ the smallest *e* value × 10, we obtained the species annotation information using LCA algorithm of MEGAN software (Huson et al., 2011; Oh et al., 2014). Based on the abundance table of species, we evaluated the similarity of samples using principal component analysis (PCA) (Avershina et al., 2013). Metastats analysis was used to identify the different species between groups (White et al., 2009).

For the functional annotations, we blasted unigenes to KEGG database (version 2018-01-01) and CAZy database (version 201801) using DIAMOND software with the parameter setting of blastp, -e 1e-5 (Kanehisa et al., 2006, 2014; Cantarel et al., 2009; Feng et al., 2015). According to the result of function annotations and the abundance table of genes, we obtained the gene number table of each sample in each taxonomy hierarchy. We also conducted the Metastats analysis of functional difference between groups.

## Results

### Summary of the Sequencing Data

A total of 114 Gb raw data were obtained from the samples. To ensure the accuracy and reliability of downstream analysis, we carried out quality control and filtering. The total amount of clean data also remained at about 114 Gb, and the average efficiency was more than 99.8% (Table 2).

**TABLE 2:** The statistical table of sequencing data.

Sample	Insert size (bp)	Raw data	Clean data	Clean_GC (%)	Effective (%)
H1	350	12,522.26	12,502.69	46.00	99.844
H2	350	12,049.48	12,022.74	45.84	99.778
H3	350	13,709.28	13,678.95	46.18	99.779
H4	350	13,407.71	13,371.57	45.87	99.730
B1	350	13,027.58	13,011.22	47.22	99.874
B2	350	13,181.78	13,165.62	47.72	99.877
B3	350	12,952.45	12,922.11	46.67	99.766
B4	350	12,593.75	12,580.36	45.22	99.894
B5	350	13,044.61	13,021.91	45.86	99.826

### Gene Prediction and Abundance Analysis

**FIGURE 1:**
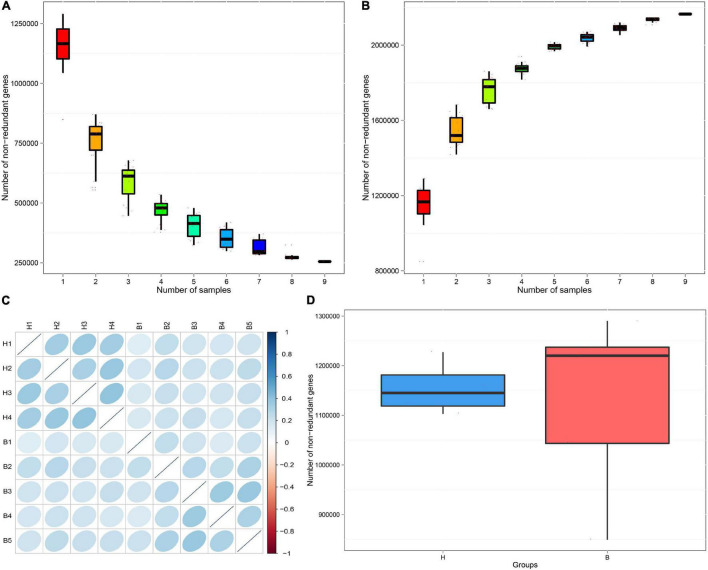
Rarefaction curves of **(A)** core genes and **(B)** pan genes. The horizontal axis represents the number of samples selected; the vertical axis represents the number of genes in the selected sample combinations. **(C)** The heatmap of correlation coefficients. The deeper the color, the greater the absolute value of the correlation between samples. **(D)** Box-plot diagram of gene number difference between groups. The horizontal axis represents the grouping information; the vertical axis represents the number of genes.

To evaluate whether the collected samples could meet the requirements of subsequent bioinformatics analysis, we conducted a rarefaction curve analysis. The results of rarefaction curve analysis based on the core genes (Figure 1A) and pan genes (Figure 1B) showed that our gene catalog captured all the available gene information in our sample. The heatmap of correlation coefficients showed that the correlation between groups is smaller than the correlation within groups, indicating reliable experimentation and reasonable sample selection (Figure 1C). From the box-plot diagram, we found that the number of genes was higher in B group compared with that in H group (Figure 1D).

### Taxonomy Prediction

Overall gut microbiota of the Mongolian gazelle comprised 141 phyla, 107 classes, 219 orders, 487 families, 1,968 genera, and 9,559 species.

**FIGURE 2:**
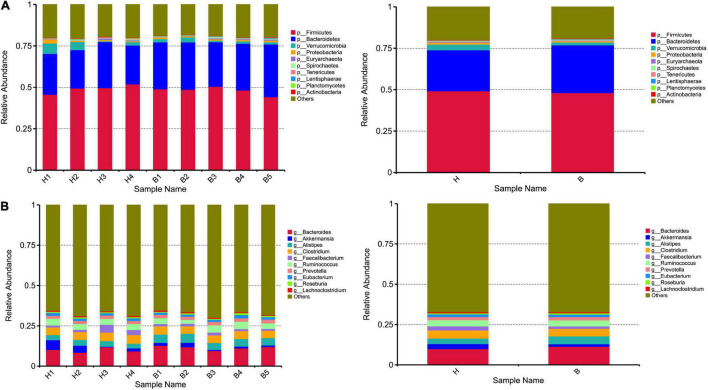
Taxonomic composition of the gut microbiota communities on the **(A)** phylum and **(B)** genus levels. Each bar represents the 10 most abundant taxa.

At the phyla level, the top five ranked abundance-based phyla in the Mongolian gazelle were Firmicutes (49.17%), Bacteroidetes (24.79%), Verrucomicrobia (3.41%), Proteobacteria (1.13%), and Euryarchaeota (0.42%) at H group; and Firmicutes (48.08%), Bacteroidetes (28.81%), Verrucomicrobia (1.71%), Proteobacteria (0.85%), and Euryarchaeota (0.42%) at B group (Figure 2A).

At the genera level, the top 10 genera are as follows: *Bacteroides*, *Akkermansia*, *Alistipes*, *Clostridium*, *Faecalibacterium*, *Ruminococcus*, *Prevotella*, *Eubacterium*, *Roseburia*, and *Lachnoclostridium* (Figure 2B).

**FIGURE 3:**
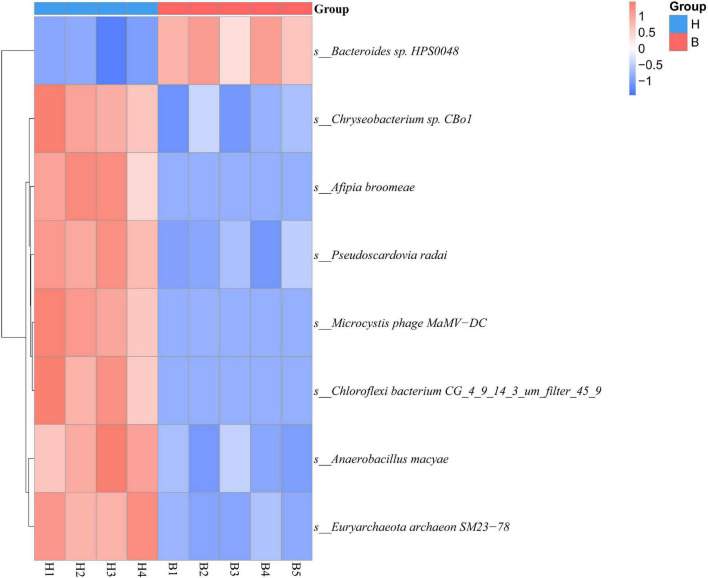
The abundance clustering based on significant differences in species at the species level. The horizontal axis represents sample information; the vertical axis represents annotated information of species; the cluster tree on the left is the species cluster tree. The values corresponding to the intermediate heat map are the *Z* values of the relative abundance of each row of species after standardized treatment.

Human exploitation and destruction of resources are currently threatening innumerable wild animal species, altering natural ecosystems and, thus, food resources, with profound effects on gut microbiota (Barelli et al., 2020). To assess the differences in gut microbiota as affected by the anthropogenic disturbance, we applied the Metastats analysis. H group and B group showed significant differences (*q* < 0.05) in the following species: *Bacteroides* sp. HPS0048, *Chryseobacterium* sp. CBo1, *Afipia broomeae*, *Pseudoscardovia radai*, *Microcystis* phage MaMV–DC, *Chloroflexi bacterium* CG_4_9_14_3_um_filter_45_9, *Anaerobacillus macyae*, and *Euryarchaeota archaeon* SM23–78 (Figure 3). PCA at the species level showed that H group and B group have distinct cluster regions (ANOSIM: *p* = 0.014, *R* = 0.444) (Supplementary Figure 1).

It is remarkable that the gut microbiota did not differ significantly between H group and B group at the both phylum and genus level. Anthropogenic disturbance only caused some changes in the gut microbiota of Mongolian gazelle at the species level.

### Common Functional Database Annotations

**FIGURE 4:**
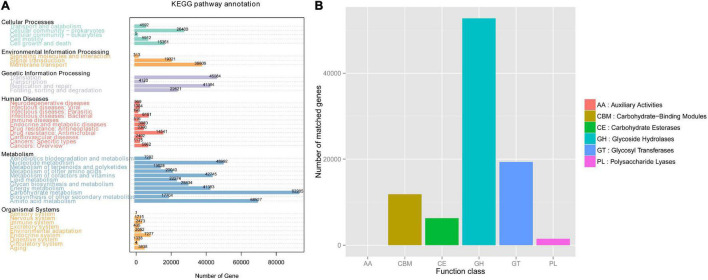
Common functional database annotations. **(A)** KEGG annotations and **(B)** CAZy annotations.

To explore the activity of genes in gut microbiota, we annotated the functional and metabolic pathways based on the KEGG and CAZy databases (Figure 4).

**FIGURE 5:**
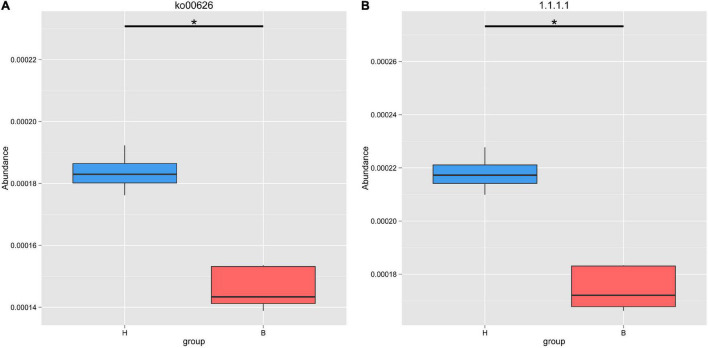
Metastats analysis of naphthalene degradation **(A)** and alcohol dehydrogenase **(B)** based on KEGG database. *Means that a significant difference was found.

Based on the KEGG database, we found that the gut microbiota of Mongolian gazelle has enriched activity for metabolism of carbohydrates (gene number: 92205), amino acid (68527), nucleotide (48992), and cofactors and vitamins (42745) (Figure 4A). The third level of KEGG classification indicated that the relative abundance of naphthalene degradation (ko00626) was significantly higher in H group than in B group (*q* < 0.05) (Figure 5A). Through Metastats analysis, we found that the alcohol dehydrogenase (EC:1.1.1.1) was significantly higher in H group than in B group (*q* < 0.05) (Figure 5B). The enzyme is mainly involved in biological processes, such as fatty acid degradation; glycine, serine, and threonine metabolism; microbial metabolism in diverse environments; and biosynthesis of secondary metabolites. For the H group, alcohol dehydrogenase was related to naphthalene degradation (Supplementary Figure 2).

**FIGURE 6:**
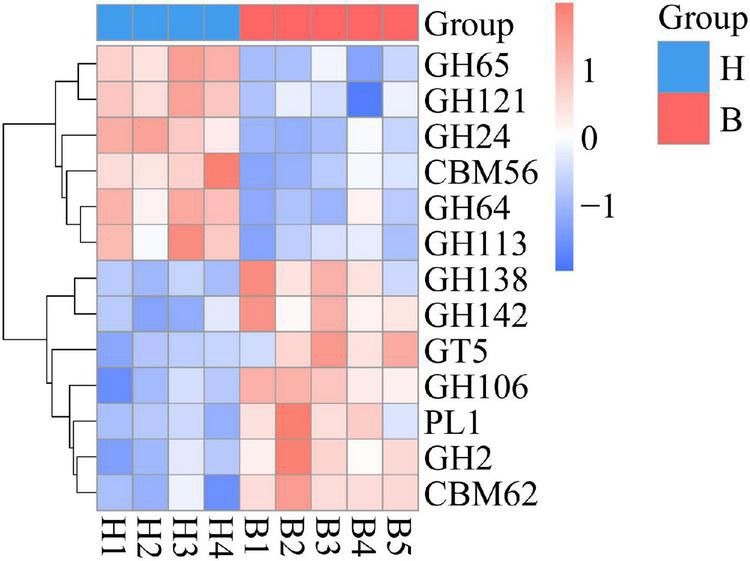
Metastats analysis in the second classification level of the CAZy database.

From the first level of CAZy classification, we found that most of the genes were annotated to the three functional configurations: glycoside hydrolases (GHs), glycosyltransferases (GTs), and carbohydrate-binding modules (CBMs) (Figure 4B). At the second classification level, GH2 (B group: 0.28%; H group: 0.24%), GT2 (B group: 0.23%; H group: 0.24%), and GH43 (B group: 0.21%; H group: 0.19%) were the most abundant enzyme families in gut microbiota of the Mongolian gazelle (Supplementary Figure 3). Among families, 6 were significantly more abundant in H group than in B group (*q* < 0.05), including GH65/121/24/64/113 and CBM56; 7 were significantly more abundant in B group than in H group (*q* < 0.05), including GH138/142/106/2, GT5, polysaccharide lyase family 1 (PL1), and CBM62 (Figure 6). At the third classification level, a total of 35 enzymes showed significant differences (*q* < 0.05) (Supplementary Figure 4). Twenty-eight enzymes were significantly more abundant in B group than in H group, including 22 enzymes of GH, 5 enzymes of GT, and 1 enzyme of PL; 7 enzymes of GH were significantly more abundant in H group than in B group.

## Discussion

Complete habitats offer wild animal inhabitants a more diverse diet than fragmented habitats. Furthermore, it has been suggested that dietary variation promotes the changes in gut microbiota (Clarke et al., 2014; Corlett, 2016). In this study, we compared the differences in gut microbiota of the Mongolian gazelle in the different habitat types. Our research showed that Firmicutes and Bacteroidetes were the prominent phyla in the Mongolian gazelle, which is consistent with previous studies of other ruminants (Sundset et al., 2007; Guan et al., 2017; Li et al., 2017). Firmicutes can degrade fibers into short-chain fatty acids, while the main functions of Bacteroidetes are to degrade fats, proteins, and carbohydrates (Jami et al., 2013). Ruminants rely on gut microbiota to harness energy by fermenting dietary material (Thoetkiattikul et al., 2013). Hence, the higher abundance of Firmicutes and Bacteroidetes can be correlated with the dietary choices of Mongolian gazelle. At the species level, we found that *Bacteroides* sp. HPS0048 was significantly more abundant in B group than in H group. The higher abundance of *Bacteroides* might indicate a healthier gut microecosystem because it can promote the improvement of immune system and maintain the balance of gut microbiota (Hooper et al., 2001; Hooper, 2004; Sears, 2005). We observe that the habitat fragmentation had no significant effect on the gut microbiota of Mongolian gazelle at both the phylum and genus level. The core gut microbiota of Mongolian gazelle at the different habitat types was relatively stable, suggesting that the effects of habitat fragmentation on gut microbiota were limited. Previous studies on ruminants indicated that 32 species had relatively stable core gut microbiota regardless of variations in dietary choices (Henderson et al., 2016). These results all indicated that ruminants had relatively stable core gut microbiota, and environment might affect the abundance of gut microbiota (Weimer, 2015).

To further understand the environmental adaptations of Mongolian gazelle, we carried out the functional analyses of gut microbiota. From the KEGG analyses, it was obvious that the metabolic pathway of naphthalene degradation has higher abundance in H group. Naphthalene belongs to polycyclic aromatic hydrocarbons and is one of the most prevalent contaminants (Mihelcic and Luthy, 1991; Ghoshal et al., 1996; Marx and Aitken, 1999). Previous studies have shown that the gut microbiota of snails can degrade naphthalene (Hu Z. et al., 2018). Given that H group is more affected by anthropogenic disturbance, we speculate that the higher abundance of naphthalene degradation is essential to maintain the stability of gut microbiota in the Mongolian gazelle.

Based on the analysis of CAZy database, we found that the GH families and CBM families have larger proportion differences between the two populations of Mongolian gazelle. The relative abundance of GH65/121/24/64/113 and CBM56 were higher in H group, whereas GH138/142/106/2 and CBM62 were higher in B group. GH families can facilitate the hydrolysis of cellulose so that Mongolian gazelle had stronger capacity for fiber digestion (Hess et al., 2011). CBM families do not show enzymatic activity, but can help GH families bind to polysaccharides and strengthen their activity (Du et al., 2018; Bernardes et al., 2019). GH families and CBM families of Mongolian gazelle can help them maximize energy from the cellulose, ensuring them to adapt to the wild environments. Our data also showed that PL1 and GT5 were significantly more abundant in B group than in H group. PL1 can cleave glycosidic bonds of homogalacturonan (HG), which is a multifunctional pectic polysaccharide of plant cell walls, and GT families can use sugar donors containing nucleoside phosphate or lipid phosphate leaving groups to catalyze the formation of glycosidic bonds (Willats et al., 2001; Lairson et al., 2008; Abbott et al., 2013). At the third classification level, we also observed that five enzymes belonging to GT5 (UDP-Glc: alpha-1,4-glucan synthase, ADP-Glc: starch glucosyltransferase, UDP-Glc: alpha-1,3-glucan synthase, UDP-Glc: glycogen glucosyltransferase, and NDP-Glc: starch glucosyltransferase) were significantly more abundant in B group than in H group. Therefore, the high abundance of PL1 and GT5 in B group showed their stronger ability to absorb carbohydrates.

## Conclusion

This study characterized the gut microbiota of Mongolian gazelle under fragmented habitats using metagenomics sequencing. Compositions of gut microbiota were similar between H group and B group, but the functions of gut microbiota differed. In the complete habitat, the high relative abundance of PL1 and GT5 promoted the absorption of carbohydrates. The higher abundance of naphthalene degradation in H group could enable Mongolian gazelle to maintain the stability of gut microbiota in the habitat that was more affected by anthropogenic disturbance. After comparison, we speculated that the variation in functions of gut microbiota in fragmented habitat might be from anthropogenic disturbance. Therefore, we recommended that minimal human disturbance and complete habitats were crucial for the survival of Mongolian gazelle.

## Data Availability Statement

The datasets generated for this study can be found in the SRA database of NCBI, accession numbers: H1 (SRR17049904), H2 (SRR17049903), H3 (SRR17049902), H4 (SRR17049901), B1(SRR17049900), B2 (SRR17049899), B3 (SRR17049898), B4 (SRR17049897), and B5 (SRR17049896).

## Ethics Statement

The animal study was reviewed and approved by the Qufu Normal University Institutional Animal Care and Use Committee.

## Author Contributions

HZ, XY, and LS conceived and designed the study. LS, XY, HD, TL, LW, SZ, YS, and YD performed the research. LS and XY analyzed the data and prepared the manuscript. All authors read and approved the final article.

## Conflict of Interest

The authors declare that the research was conducted in the absence of any commercial or financial relationships that could be construed as a potential conflict of interest.

## Publisher’s Note

All claims expressed in this article are solely those of the authors and do not necessarily represent those of their affiliated organizations, or those of the publisher, the editors and the reviewers. Any product that may be evaluated in this article, or claim that may be made by its manufacturer, is not guaranteed or endorsed by the publisher.
