# Supplementary material for: Comparative Analysis of the Gut Microbiota of Mongolian Gazelle (Procapra gutturosa) Under Fragmented Habitats
Source: Front Microbiol. 2022 Mar 9;13:830321. doi: 10.3389/fmicb.2022.830321 (PMC8965509; doi:10.3389/fmicb.2022.830321)
Supplement: Supplementary file 1 [file Data_Sheet_1.docx]

Supplementary Material

# Supplementary Figures and Tables

**Figure S1.** Principal component analysis (PCA) of the fecal bacterial communities on the species level.

**Figure S2.** The pathway of naphthalene degradation.

**Figure S3.** The relative abundance of top10 in the second classification level of the CAZy database.

**Figure S4.** Metastats analysis in the third classification level of the CAZy database.

**Table S1.** Detailed information for all samples.

| Sample group | Collection location | Sample name | Collection time |
| --- | --- | --- | --- |
| H group | Hulun Lake National Nature Reserve | H1 | 2018/12 |
|  |  | H2 | 2019/12 |
|  |  | H3 |  |
|  |  | H4 |  |
| B group | China-Mongolia border area | B1 | 2019/12 |
|  |  | B2 |  |
|  |  | B3 |  |
|  |  | B4 |  |
|  |  | B5 |  |
