# Supplementary figures and images for: Comparative Analysis of the Gut Microbiota of Mongolian Gazelle (Procapra gutturosa) Under Fragmented Habitats
Source: Front Microbiol. 2022 Mar 9;13:830321. doi: 10.3389/fmicb.2022.830321 (PMC8965509; doi:10.3389/fmicb.2022.830321)

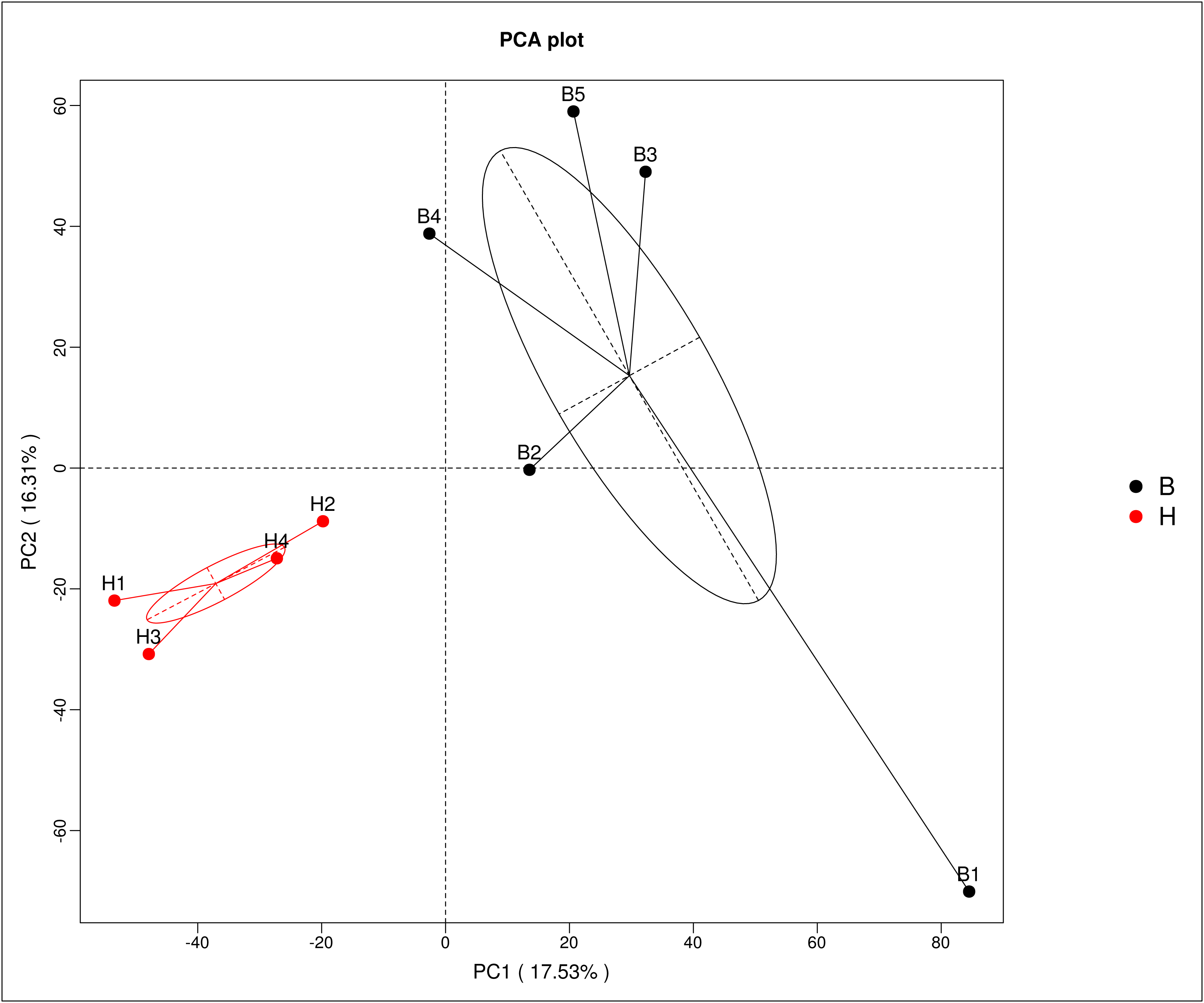

Supplement: Supplementary file 2 [file Image_1.JPEG]

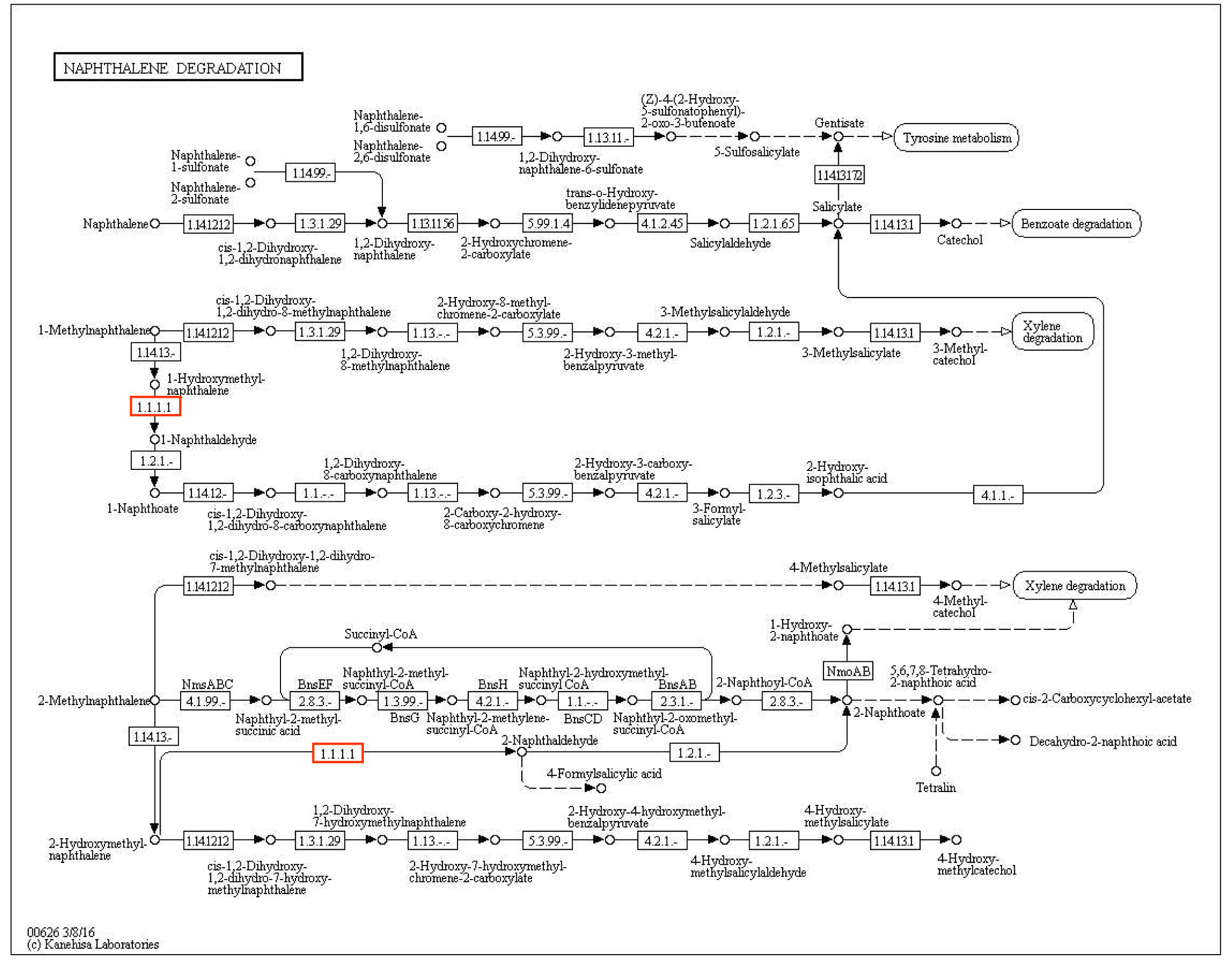

Supplement: Supplementary file 3 [file Image_2.JPEG]

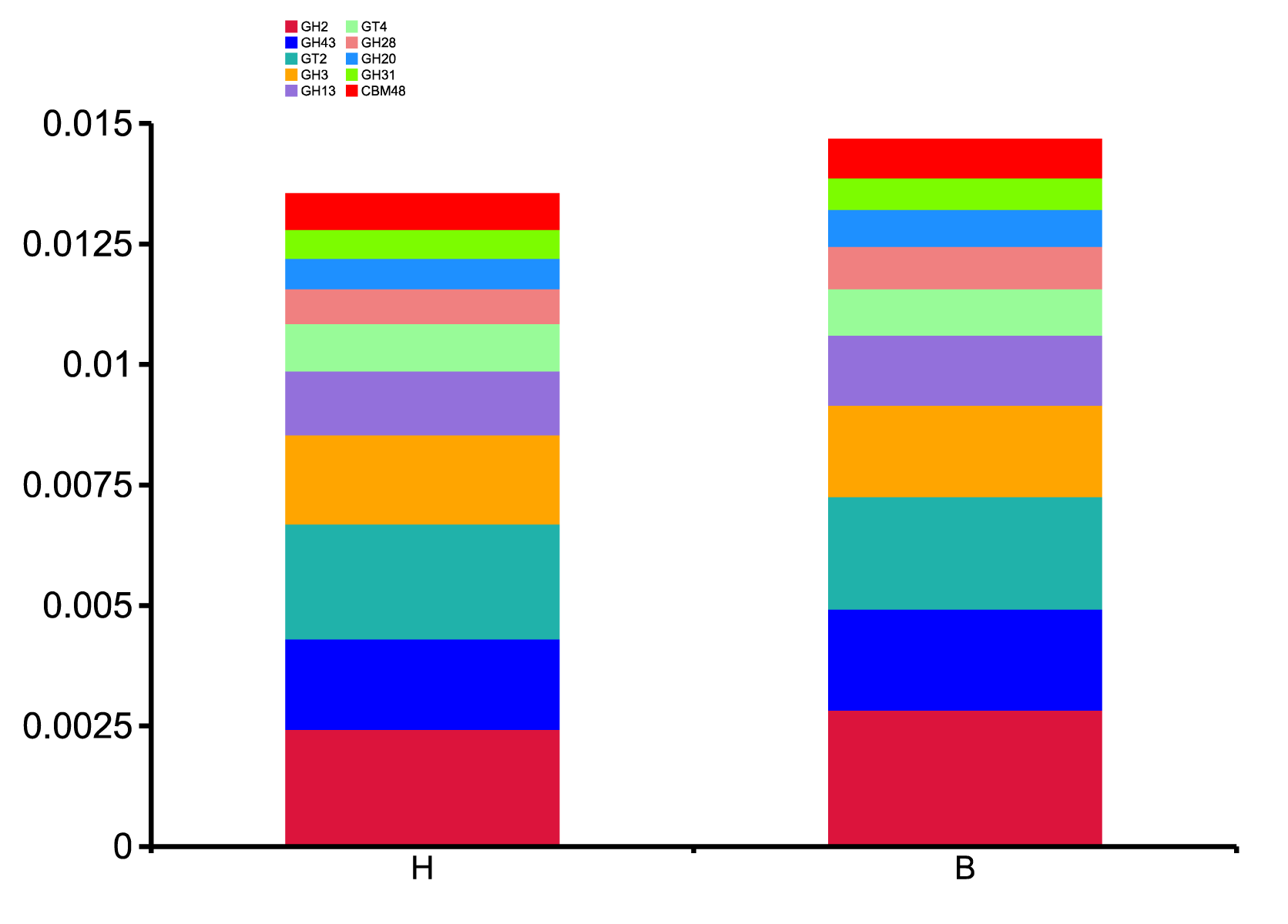

Supplement: Supplementary file 4 [file Image_3.TIF]

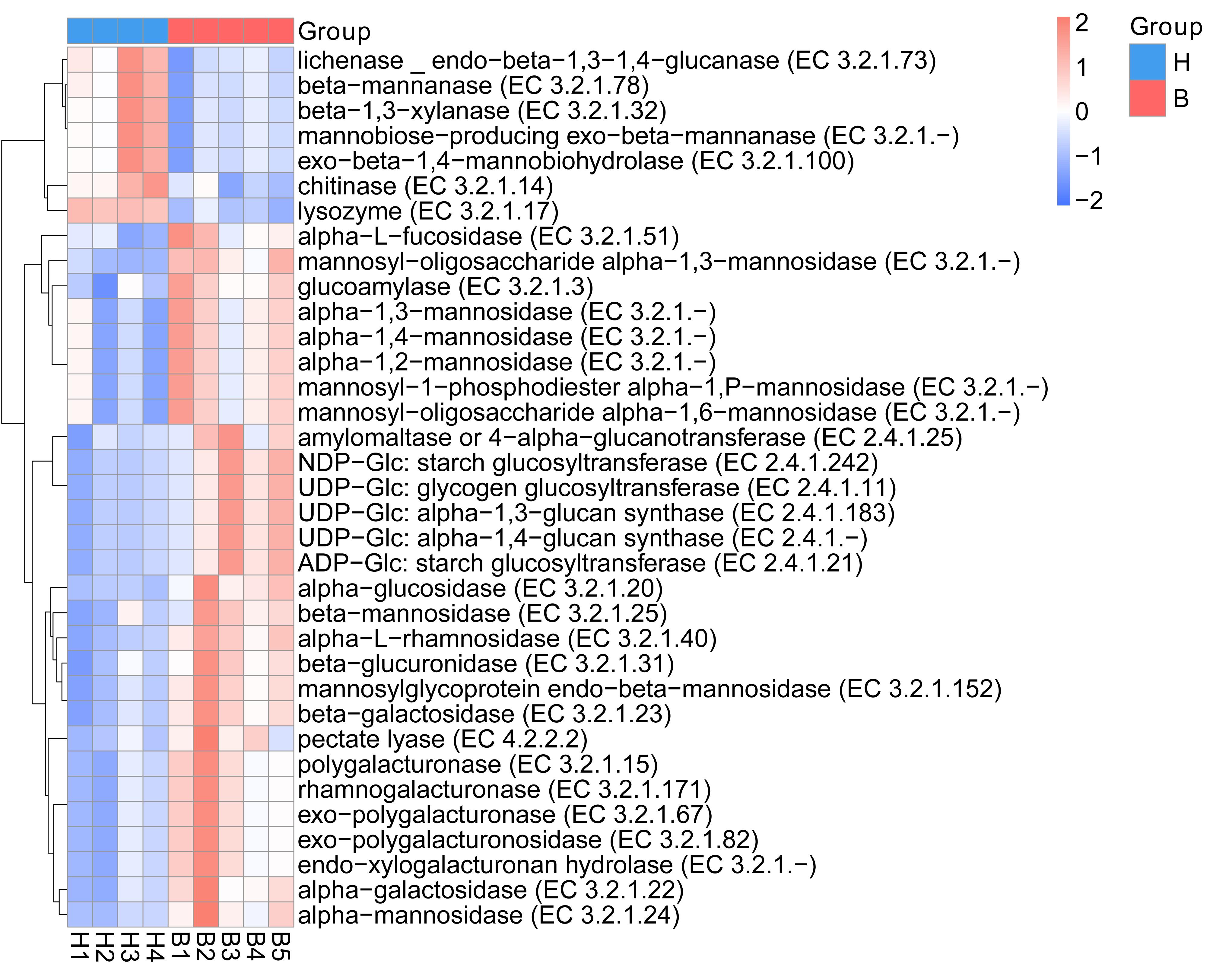

Supplement: Supplementary file 5 [file Image_4.JPEG]
